# The average 30-minute post-prandial C-peptide predicted diabetic retinopathy progress: a retro-prospective study

**DOI:** 10.1186/s12902-023-01300-x

**Published:** 2023-03-16

**Authors:** Ting Pan, Jie Gao, Xinghua Cai, Huihui Zhang, Jun Lu, Tao Lei

**Affiliations:** 1 School of Medical and Life Sciences, Chengdu University of Traditional Chinese Medicine, Chengdu, China; 2 Department of Endocrinology, Putuo Hospital, Shanghai University of Traditional Chinese Medicine, 164 LanXi Road, 200062 Shanghai, China; 3 Shanghai Putuo Central School of Clinical Medicine, Anhui Medical University, Anhui, China

**Keywords:** C-peptide, Diabetic Retinopathy, Type 2 diabetes Mellitus

## Abstract

**Background:**

The conclusion between Connecting peptide (C-peptide) and diabetic chronic complication was controversial. The purpose of this study is to explore the possible association between average C-peptide with diabetic retinopathy (DR) progression in Chinese patients with type 2 diabetes.

**Methods:**

This is a retro-prospective study. 622 patients with type 2 diabetes were included. DR was evaluated using non-mydriatic fundus photography and DR progression was defined as any deterioration of either eye. Fasting and postprandial c-peptide levels were assayed at baseline and follow-up period. Differences between continuous variables were compared using the Mann–Whitney *U* test; and categorical variables were analyzed by the chi-square test. Correlation between parameters and 30-minute postprandial C-peptide were determined by Spearman correlation test. The relationship between C-peptide and DR progression was evaluated by multivariable binary logistic regression. Two-tailed P-values < 0.05 were regarded as statistically significant.

**Results:**

DR was present in 162 (26.0%) patients at baseline, and 26.4% of patients were found progression of DR at follow-up. Patients with progression of DR had lower average levels of 30-minute postprandial C-peptide (2.01 ng/ml vs. 2.6 ng/ml, *p* = 0.015) and 120-minute postprandial C-peptide (3.17 ng/ml vs. 3.92 ng/ml, *p* < 0.029), as well as average increment of 30-minute (0.41 ng/ml vs. 0.64 ng/ml, *p* = 0.015) and 120-minute postprandial C-peptide (1.48 ng/ml vs. 1.93 ng/ml, *p* < 0.017), than those without DR aggravation. Multivariate logistic regression analysis determined that 30-minute postprandial C-peptide and its increment were related to reduced odds ratios for DR progression (odds ratios [OR] = 0.83 and 0.74, respectively).

**Conclusion:**

Our results suggest that the Average 30-minute post-prandial C-peptide and increment were negatively correlated with DR progression, which further demonstrates the importance to preserve β-cell residual function in the prevention for DR progression.

**Trial registration:**

Not applicable.

## Background

Impaired pancreatic islet β-cell compensatory function along with insulin resistance make a valuable contribution towards the occurrence of type 2 diabetes in Eastern Asia [1, 2]. In comparison to basic insulin secretion which was related to fasting plasma glucose, decreased early phase insulin secretion was associated with elevated post-load glucose [3]. Hyperglycemic clamp study indicated first phase insulin infusion inhibited hepatic glucose production by 90% [4, 5]. C-peptide is split from proinsulin, composed of 31 amino acids, and secreted in an equimolar pattern with insulin under stimulation by elevated glycemia. As a result, C-peptide was used as an alternative marker of β-cell function in clinical practice [6]. Similarly, numerous evidences indicated that C-peptide was closely associated with fasting and post-load glucose [7]. Moreover, in oral glucose tolerance test (OGTT), early and late C-peptide reactions have opposite predictive effects on type 1 diabetes in autoantibody positive individuals [8]. Recently, several studies indicated that postprandial C-peptide is a useful predictor of future insulin therapy in patients with type 2 diabetes [9–12].

The conclusion between C-peptide and chronic complication was controversial [6]. Some trials have demonstrated that C-peptide is positively correlated with macrovascular complications and negatively related to microvascular complications [6]. Diminished glucose/C-peptide ratios are associated with reduced renal function [13]. Among microvascular complications, DR affected 20% of patients with diabetes, and caused millions of new blindness cases in adults ranging 20 to 74 years old [14, 15]. Fasting C-peptide has been verified to be negatively associated with DR [15, 16]. Although researchers pointed out that post-load glucose was independently associated with urine albumin excretion, cardiovascular diseases (CVD), cancer, and mortality in the multivariable models [17, 18], the association between post-load C-peptide and DR progression was seldomly involved.

In the present study, we aimed to find out the relationship between C-peptide and DR in the Cohort including 622 type 2 diabetes patients, and to explore the connection between post-load C-peptide and DR progression.

## Methods

### Study design and patients

This retro-prospective study was performed in Putuo hospital, which is affiliated to Shanghai University of Traditional Chinese Medicine. Aim to explore the possible association between average C-peptide with DR progression in Chinese patients with type 2 diabetes. A total of 622 patients with type 2 diabetes underwent non-mydriatic fundus photography were included in our study From February 27th 2003 to April 25th 2012. Patients were excluded in the presence of any of the following conditions: (1) type 1 diabetes mellitus; (2) glaucoma, retinal detachment, vitreous hemorrhage; (3) participants with a history of intraocular surgery or had intraocular surgery at either eye during follow-up; (4) patients who lost to follow-up or had no clinical data recorded during follow-up; (5) renal dysfunction (serum creatinine ≥ 115 µmol/L), hepatic dysfunction (alanine transaminase ≥ 97.5 U/L and/or aspartate aminotransferase ≥ 55.5 U/L), hematological disease, rheumatic diseases, malignant tumors.

The protocol was approved by the institutional review board of Putuo hospital, Shanghai University of Traditional Chinese Medicine. Written informed consent was obtained from all participants in this study.

### Anthropometric and laboratory measurements

All anthropometric and biochemical examinations at baseline and follow up used the same study protocol. Measure waist circumference, weight and height according to standard procedures. The Body Mass Index (BMI) was computed using the formula of the weight divided by meters squared (kg/m^2^). After a 10-minute of calm, the subjects sat down and measured their blood pressure twice with a mercury sphygmomanometer, taking the average value. According to the ingestion of a diabetic diet based on the criteria recommended by the Chinese Diabetes Society [19], peripheral venous blood samples of all subjects were collected at 0, 30, and 120 min after at least 10-hour overnight fasting. Hemoglobin A1c (HbA1c) was detected using the HLC-723G11 (Shunan, Yamaguchi, Japan) high-performance liquid chromatography analyzer; plasma glucose concentrations were measured using the glucose oxidase method (Roche, Mannheim, Germany); an automatic analyzer (Hitachi 7600, Tokyo, Japan) was used to determine the lipid profiles; C-peptide was determined with a radioimmunoassay method (Linco Research, St Charles, MO, USA).

### Assessment of diabetic retinopathy

Retina screening was performed using non-mydriatic fundus photography by a professional ophthalmologist. The severity of DR is graded according to the 2003 standard proposed by the American Academy of Ophthalmology[20]: no apparent retinopathy DR (Grade 0); mild non-proliferative DR (Grade I); moderate non-proliferative DR (Grade II); severe non-proliferative DR (Grade III); and proliferative DR (Grade IV). DR progression was defined as participants without baseline DR who developed DR or any greater worsening of DR at any eye during the follow-up.

### Statistical analysis

Median (interquartile range) were presented for continuous variables, while frequency (%) for categorical variables. Differences between continuous variables were compared using the Mann–Whitney *U* test; and categorical variables were analyzed by the chi-square test. Correlation between parameters and 30-minute postprandial C-peptide were determined by Spearman correlation test. The relationship between C-peptide and DR progression was evaluated by multivariable binary logistic regression. SPSS 25.0 (SPSS Inc., Chicago, IL, USA) software was used for all statistical analysis. Two-tailed P-values < 0.05 were regarded as statistically significant.

## Results

### Baseline clinical characteristics of participants according to DR status

Baseline clinical characteristics of all patients were listed in Table 1. Overall, 162 (26.0%) patients had DR at baseline. Compared to patients without DR, patients with DR were older (60 years versus 57 years, P < 0.05), had longer diabetes duration (10 years versus 4 years, P < 0.001) and higher frequency of insulin therapy. Levels of 120-minute postprandial C-peptide (PCP) and its increment were lower in patients with DR than those without DR. The prevalence of males, hypertension, smoking and alcohol consumption were similar in the two groups. Moreover, there were no difference in BMI, waist circumference (WC), blood pressure, levels of fasting blood glucose, postprandial blood glucose, HbA1c, lipid level, fasting C-peptide, 30-minute PCP and its increment.

### Clinical features during follow-up according to the DR progression status

As shown in Table 2, DR progression was detected in 164 (26.4%) of the subjects at follow-up. Patients with DR progression had higher average HbA1c levels (8.9% versus 8.5%, P < 0.05), and lower levels of average 30-minute PCP (median 2.01 ng/ml versus 2.60 ng/ml, P = 0.015), average 120-minute PCP (median 3.17 ng/ml versus 3.92 ng/ml, P = 0.029), average 30-minute PCP increment (median 0.41 ng/ml versus 0.64 ng/ml, P = 0.015) and average 120-minute PCP increment (median 1.48 ng/ml versus 1.93 ng/ml, P = 0.017). The two groups had similar levels of average waist circumstance, blood pressure, blood lipid, fasting and post prandial plasma glucose as well as fasting plasma C-peptide.

### Clinical characteristics correlating with 30-minute postprandial C-peptide

As shown in Table 3, average 30-minute PCP as well as its increment was moderately associated with average HbA1c, body weight, WC, BMI, diastolic blood pressure (DBP), High-density lipoprotein cholesterol (HDL-C), triglyceride and total cholesterol (all *P* < 0.05). There were non-significant associations of average 30-minute PCP with average fasting plasma glucose, average 30-minute post-prandial glucose (PPG) and 120-minute PPG.

### Odds ratios for DR progression according to C-peptide levels

As displayed in Table 4, multivariate logistic regression analysis indicated that 30-minute postprandial C-peptide and its increment were significantly related to DR progress risk reduction (OR = 0.83 and 0.74, respectively), this association remained significant after further adjusted for diabetes management including insulin therapy and insulin secretagogue. However, the association of baseline fasting C-peptide (FCP), 30-minute PCP, 120-minute PCP, and average FCP and 120-minute PCP with DR progression was non-significant after adjusting confounding variables.

**Table 1:** Baseline characteristics of patients

Variables	No DR (n = 460)	DR (n = 162)	P value
Gender (men), %	276 (60.0)	93 (57.4)	0.56
Age, years	57 (47–68)	60 (52–70)	0.047
Diabetes course, years	4.0 (1.0–9.0)	10.0 (4.8–15.0)	< 0.001
BMI, kg/m^2^	24.3 (22.3–26.8)	25.0 (22.0–27.5)	0.56
WC, cm	89.5 (81.0–96.0)	89.5 (84.0–97.0)	0.90
Current smoking, %	144 (31.3)	40 (24.8)	0.12
Alcohol drinking, %	72 (15.7)	24 (14.9)	0.82
HTN, %	250 (54.7)	98 (60.5)	0.20
SBP, mmHg	130 (120–140)	130 (120–145)	0.29
DBP, mmHg	80 (70–85)	80 (75–85)	0.98
HbA1c, %	8.5 (7.0–10.5)	8.95 (7.5–10.6)	0.08
FPG, mmol/L	8.33 (6.80–10.76)	8.98 (6.80- 11.94)	0.36
30-minute PPG, mmol/L	7.49 (5.80–9.11)	7.45 (5.85–9.36)	0.90
120-minute PPG, mmol/L	13.38 (10.52–16.47)	13.29 (10.55–17.26)	0.99
FCP, ng/ml	1.78 (0.99–2.80)	1.52 (0.84–2.53)	0.09
30-minute *PCP*, ng/ml	2.72 (1.37–4.32)	2.17 (1.19–3.44)	0.10
120-minute PCP, ng/ml	3.94 (2.06–6.25)	3.16 (1.66–5.30)	0.015
TC, mmol/L	4.80 (4.10–5.50)	4.80 (4.30–5.40)	0.77
TG, mmol/L	1.59 (1.05–2.34)	1.57 (1.04–2.33)	0.84
HDL-C, mmol/L	1.08 (0.93–1.33)	1.12 (0.96–1.42)	0.26
LDL-C, mmol/L	2.96 (2.44–3.61)	2.97 (2.51–3.48)	0.834
30-minute PCP increment, ng/ml	0.60 (0.20–1.30)	0.40 (0.10–1.00)	0.14
120-minute PCP increment, ng/ml	2.00 (0.60–3.70)	1.40 (0.50–2.80)	0.006
Diabetes management			
Insulin, n (%)	282 (61.3)	115 (71)	0.027
insulin secretagogues, n (%)	111 (24.1)	35 (21.6)	0.51

Data were expressed as median (interquartile range, IQR)

Abbreviations: *FPG*, fasting plasma glucose; *PPG*, post-prandial glucose; *FCP*, Fasting C- peptide; *PCP*, post-prandial C- peptide; *SBP*, systolic blood pressure; *DBP*, diastolic blood pressure; *BMI*, body mass index; *WC*, waist circumstance; *TC*, total cholesterol; *HTN*, hypertension; *TG*, triglyceride; *HDL-C*, high-density lipoprotein cholesterol; *LDL-C*, low-density lipoprotein cholesterol

**Table 2:** Clinical characteristics according to diabetic retinopathy progression during follow-up

Variables	No DR progression (n = 458)	DR progression (n = 164)	*P* value
Follow-up period, years	2.5 (1.5–3.9)	3.0 (1.9–4.1)	0.027
Gender (men), %	272 (59.4)	99 (59.1)	0.96
Average BMI, kg/m^2^	24.5 (22.2–26.9)	24.9 (23.3–27.2)	0.12
Average WC, cm	90 (81.5–95.6)	90.5 (85–98)	0.22
Average SBP, mmHg	130 (120–140)	133 (121–143)	0.11
Average DBP, mmHg	80 (75–85)	80 (75–85)	0.97
Average HbA1c, %	8.5 (7.3–9.81)	8.9 (7.8–10.1)	0.031
Average FPG, mmol/L	8.30 (6.89–10.2)	8.86 (7.10–10.9)	0.10
Average 30-minute PPG, mmol/L	7.45 (5.94–9.19)	7.43 (5.67–9.53)	0.90
Average 120-minute PPG, mmol/L	13.3 (10.9–15.8)	13.75 (11.1–15.8)	0.31
Average FCP, ng/ml	1.76 (0.97–2.54)	1.52 (0.84–2.44)	0.19
Average 30-minute PCP, ng/ml	2.60 (1.37–3.82)	2.01 (1.19–3.43)	0.015
Average 120-minute PCP, ng/ml	3.92 (2.20–5.72)	3.17 (1.76–5.19)	0.029
Average TC, mmol/L	4.70 (4.06–5.31)	4.8 (4.36–5.30)	0.10
Average TG, mmol/L	1.49 (1.02–2.25)	1.6 (1.08–2.21)	0.33
Average HDL-C, mmol/L	1.09 (0.96–1.33)	1.11 (0.98–1.35)	0.83
Average LDL-C, mmol/L	2.99 (2.51–3.5)	3.05 (2.51–3.54)	0.54
Average 30-minute PCP increment, ng/ml	0.64 (0.15–1.35)	0.41 (0.06–0.99)	0.015
Average 120-minute PCP increment, ng/ml	1.93 (0.85–3.36)	1.48 (0.58–2.71)	0.017
DR at baseline, %	83 (18.1)	79 (48.2)	< 0.001

Abbreviations: *FPG*, fasting plasma glucose; *PPG*, post-prandial glucose; *FCP*, Fasting C peptide; *PCP*, post-prandial C peptide; *SBP*, systolic blood pressure; *DBP*, diastolic blood pressure; *BMI*, body mass index; *WC*, waist circumstance; *TC*, total cholesterol; *HTN*, hypertension; *TG*, triglyceride; *HDL-C*, high-density lipoprotein cholesterol; *LDL-C*, low-density lipoprotein cholesterol; *HbA1c*, Hemoglobin A1c

**Table 3:** Parameters correlated with 30-minute post-prandial C- peptide

	Average 30-min PCP			Average 30-min PCP increment	
	r	P value		r	P value
Average body weight, kg	0.364	< 0.001		0.241	< 0.001
Average BMI, kg/m^2^	0.426	< 0.001		0.246	< 0.001
Average WC, cm	0.355	< 0.001		0.196	< 0.001
Average SBP, mmHg	0.054	0.18		-0.001	0.97
Average DBP, mmHg	0.098	0.015		0.046	0.25
Average HbA1c, %	-0.305	< 0.001		-0.232	< 0.001
Average FPG, mmol/L	-0.07	0.09		0.108	0.007
Average 30-minute PPG, mmol/L	-0.056	0.19		-0.089	0.036
Average 120-minute PPG, mmol/L	0.036	0.37		0.033	0.41
Average TG, mmol/L	0.354	< 0.001		0.202	< 0.001
Average HDL-C, mmol/L	-0.343	< 0.001		-0.203	< 0.001
Average TC, mmol/L	0.079	0.049		0.043	0.29
Average LDL-C, mmol/L	0.077	0.06		0.047	0.24

Abbreviations: *FPG*, fasting plasma glucose; *PPG*, post-prandial glucose; *SBP*, systolic blood pressure; *DBP*, diastolic blood pressure; *BMI*, body mass index; *WC*, waist circumstance; *TC*, total cholesterol; *TG*, triglyceride; *HDL-C*, high-density lipoprotein cholesterol; *LDL-C*, low-density lipoprotein cholesterol; *HbA1c*, Hemoglobin A1c

.

**Table 4:** Association between C peptide and diabetic retinopathy progression

Variables	Model 1			Model 2	
	OR (95% CI)	P value		OR (95% CI)	P value
Baseline FCP, ng/ml	0.91 (0.77–1.08)	0.31		0.92 (0.77–1.09)	0.32
Baseline 30-min PCP, ng/ml	0.91 (0.79–1.04)	0.18		0.91 (0.79–1.05)	0.21
Baseline 120-min PCP, ng/ml	1.02 (0.96–1.08)	0.61		1.02 (0.96–1.08)	0.54
Average FCP, ng/ml	0.86 (0.69–1.06)	0.16		0.86 (0.69–1.07)	0.17
Average 30-min PCP, ng/ml	0.83 (0.72–0.97)	0.018		0.83 (0.71–0.97)	0.018
Average 120-min PCP, ng/ml	0.99 (0.91–1.08)	0.81		0.99 (0.91–1.08)	0.89
Average 30-min PCP increment, ng/ml	0.74 (0.57–0.95)	0.020		0.74 (0.57–0.96)	0.021
Average 120-min PCP increment, ng/ml	1.02 (0.92–1.13)	0.69		1.03 (0.93–1.14)	0.61

Model 1: adjusted by gender, age at baseline, smoking status, alcohol drinking, baseline DR status, hypertension, follow-up period, average levels of waist circumstance, HbA1c, triglyceride and HDL-C; Model 2: further adjusted for diabetes management including insulin therapy and insulin secretagogue

## Discussion

The present study indicated that in patients with type 2 diabetes, levels of postprandial C-peptide and its increment were lower in patients with DR. Furthermore, 30-minute postprandial C-peptide concentrations and its increment were significantly connected with reduced risk of DR progression. This relationship remained significant after adjusting for confounding risk factors including gender, age, smoking status, alcohol drinking, baseline DR status, hypertension, follow-up period, HbA1c, triglyceride and HDL-C.

DR is the main reason for new cases of blindness in adults aged 20–74 [14]. The DR prevalence of diabetes patients obtained from different studies have shown substantial variability, ranging from 17.6% in an India study [21] to 33.2% in a large U.S. study [22]. A recent report [23] showed that the prevalence of DR in diabetic group was 23.57% in China. In our study, the frequency of DR was 26.0% at baseline.

The link between C-peptide and DR in patients with type 2 diabetes is still controversial. Several previous studies have shown that lower levels of C-peptide are negatively correlated with DR in Type 2 Diabetes Mellitus (T2DM) patients, indicating that C-peptide has a protective effect on microvascular complications of diabetes [24–27], while other studies did not find the association [28, 29]. The study from Korea demonstrated that patients with lower C-peptide increment (postprandial –fasting C-peptide) had a higher risk of DR and an increased DR severity [27]. In addition, other research studies have shown that the lower postprandial C-peptide concentration may be related to the higher incidence rate of DR. Our results are in agreement with those studies that postprandial C-peptide levels and its increment are negatively associated with DR [23–27]. However, in Australian population, systolic blood pressure, HbA1c and duration of diabetes are independent risk factors for the development of DR, not C-peptide [30]. In this prospective cohort, our data verified that average 30-minute post-prandial C-peptide and its increment was negatively correlated with DR progression.

The relationship between C-peptide and DR was complicated. First, C-peptide reflects the endogenous insulin secretion of diabetes patients [31, 32] and is related to the improvement of blood glucose control and the reduction of blood glucose variability [24, 33–36], which is conducive to reducing DR risk. Besides, C-peptide can regulate insulin secretion, islet microcirculation and glucose tolerance in rats [37]. Secondly, studies have reported that C-peptide may have its own biological activity and exert some physiological function [6]. Studies have demonstrated that C-peptide may have a direct impact on the development and/or progression of microvascular complications [38, 39]. Thirdly, C-peptide could inhibit the vascular leakage induced by hyperglycemia in the diabetic retina and suppress the function of vascular endothelial growth factor (VEGF), which is a crucial factor that leads to elevated retinal microvascular permeability in diabetes and promotes the progression of DR [40, 41]. In addition, C-peptide could reduce neointimal hyperplasia induced by insulin [42].

Nevertheless, several limitations of our study should be taken into account. First, patients included in this study were from a single center and the sample could not represent the entire patient population. Second, the follow-up period was relatively short and further analysis with a longer follow-up is required. Third, other confounding factors such as osteoporosis may also be considered.

## Conclusion

The average 30-minute post-prandial C-peptide and its increment were negatively associated with DR progression, demonstrating the importance of preserving β-cell residual function in the prevention for DR progression. Better residual β-cell function may partly explain the lower risk of DR in patients with type 2 diabetes.

## Data Availability

The datasets during the current study are available from the corresponding author on reasonable request.

## Abbreviations

BMI: Body Mass Index
C-peptide: Connecting peptide
CVD: Cardiovascular diseases
DR: Diabetic retinopathy
DBP: Diastolic blood pressure
FPG: Fasting plasma glucose
FCP: Fasting C-peptide
HbA1c: Hemoglobin A1c
HDL-C: High-density lipoprotein cholesterol
HTN: Hypertension
kg/m^2^: Meters squared
OGTT: Oral glucose tolerance test
PCP: Postprandial C-peptide
PPG: Post-prandial glucose
SBP: Systolic blood pressure
T2DM: Type 2 Diabetes Mellitus
TG: Triglyceride
TC: Total cholesterol
VEGF: Vascular endothelial growth factor
WC: waist circumference
